# LBW and IUGR temporal trend in 4 population-based birth cohorts: the role of economic inequality

**DOI:** 10.1186/s12887-016-0656-0

**Published:** 2016-07-29

**Authors:** Ana D. I. Sadovsky, Alicia Matijasevich, Iná S. Santos, Fernando C. Barros, Angelica E. Miranda, Mariangela F. Silveira

**Affiliations:** 1 Department of Pediatrics, Federal University of Espírito Santo (UFES), Marechal Campos Ave., 1468 – Maruipe, Vitória, ES Zip code: 29040-091 Brazil; 2 Postgraduate Program in Epidemiology, Federal University of Pelotas (UFPel), Pelotas, RS Brazil; 3 Department of Preventive Medicine, University of São Paulo (USP), São Paulo, SP Brazil; 4 Postgraduate Program in Health and Behavior, Catholic University of Pelotas, Pelotas, RS Brazil; 5 Postgraduate Program in Public Health, Federal University of Espírito Santo (UFES), Vitória, ES Brazil

**Keywords:** Inequality, Income, Preterm, Low birthweight, Small for the gestational age, Intrauterine growth restriction, Poverty

## Abstract

**Background:**

Low/medium income countries, with health inequalities present high rates of neonates having low birthweight and/or are small for the gestational age. This study aims to analyze the absolute and relative income inequality in the occurrence of low birthweight and small size for gestational age among neonates in four birth cohorts from southern Brazil in 1982, 1993, 2004, and 2011.

**Methods:**

The main exhibit was monthly family income. The outcomes were birth with low birthweight or small for the gestational age. The inequalities were calculated using the Slope Index of Inequality and the Relative Index of Inequality adjusted for maternal skin color, schooling, age, and marital status.

**Results:**

In all birth cohorts, poorer mothers were at greater odds of having neonates with low birthweight or small for the gestational age. There was a tendency to decrease the prevalence of small for gestational age in poorer families associated with the reduction of inequalities over the past decades, which was not observed regarding low birthweight.

**Conclusions:**

Economic inequalities occurred in neonates with low birthweight and with intrauterine growth restriction in the four studies, with a higher incidence of inadequate neonatal outcomes in the poorer families.

## Background

Socioeconomic and demographic inequalities remain a great challenge for healthcare strategies or policies in countries of low and medium income [1]. The factors that contribute to these inequalities may be related to gender, skin color, schooling, wealth, employment access to healthcare, among others [2] which impact the socioeconomic level of the individuals in society, with continuous and gradual effects on health throughout their lives [3].

Underprivileged regions regarding economy and schooling that have great inequalities in healthcare, particularly for mothers and infants, have high rates of worrisome neonatal outcomes such as intrauterine growth restriction (IUGR), which is manifested as neonates (NN) with low birthweight (LBW) or small for the gestational age (SGA) [4, 5]. These outcomes contribute to higher child morbidity-mortality and are relevant to the making of health policies especially in low- and medium-income countries [5–7].

The present study aims to analyze the pattern of prevalence of neonates with low birthweight and intrauterine growth restriction (IUGR), in four birth cohorts in Pelotas, RS, southern Brazil, in 1982, 1993, 2004, and 2011 according to the absolute and relative inequality in family income.

## Methods

### Research setting and study design

Four birth cohort studies were carried out in the city of Pelotas. Throughout 1982, 1993, and 2004, all births in hospitals were identified and those whose mothers lived in the urban area of Pelotas were included in the cohorts. Soon after birth, the mothers were interviewed using a previously tested structured questionnaire and a specially trained team under the supervision of a pediatrician examined the NN. Anthropometric measures were taken from the mothers and their NN. Methodological details of each cohort (1982, 1993, and 2004) were described in previous publications [8–10].

The data from 2011 came from the multi-center study International Fetal and Newborn Growth Consortium for the 21st Century – Intergrowth 21st, in which the city of Pelotas represents Latin America. The inclusion criteria, the sampling, logistics, and implementation were similar to those of the cohorts described above and can be found in the publications by Villar et al. (2013) and Silveira et al. (2013) [11, 12].

### Outcomes and covariates

The following outcome variables were studied: (i) LBW, when weight at birth < 2,500 g [13] and (ii) IUGR, when the NN’s weight was below the 10th percentile in the standard weight curve by sex and gestational age (SGA) [13], according to Williams growth curve, recommended by the World Health Organization [14]. In the study of 1982 and 1993, the gestational age was evaluated by Dubowitz’s method through neonates’ physical examination in the first day of life [9, 15]. In 2004 study, gestational age was assessed by the first day of the last menstrual period (LMP), by ultrasound (when available) and by Dubowitz’s method [8]. In 2011 study, gestational age was estimated by a combination of LMP, ultrasound when available and neonatal measures using an internationally standard chart. [12].

The main exposure was monthly family income calculated from the sum of the individual incomes collected continuously. Later, the income was categorized as income quintiles. The exposure variables were mother’s skin color, categorized as white, black, or others by the interviewer, except in the 2011 study, in which it was self-reported; mother’s schooling in full years, expressed as five categories: < 4 years, 5 to 8 years, 9 to 11 years, and ≥ 12 years; mother’s age in full years categorized into < 20 years, 20 to 34 years, and ≥ 35 years; and mother’s marital status, expressed as whether she was living with a partner or not regardless of the legal status of the marriage.

### Inequality indices

There are several methods to measure inequalities, with different complexity levels, and their choice depends on the goal of the study. The slope index of inequality (SII) and the relative index of inequality (RII) have been used to score the magnitude of the absolute and relative differences, respectively, of socioeconomic level indicators. These indices are used to compare temporal trends of neonatal outcomes in epidemiologic studies [2].

SII is obtained from the regression of the outcome in healthcare (LBW and SGA NN) in the mean of the relative income rank with values ranging between 0 and 1. Initially, to calculate the relative income rank, the income quintiles were sorted from the lowest to the highest. Later, the mean point of the distribution in this rank was calculated. Each mean point of each quintile was located at 0.1, 0.3, 0.5, 0.7, and 0.9, approximately. SII was obtained from the regression of each outcome of the mean point in the rank and was interpreted as the absolute difference in the outcome between the individual at the lowest point of the distribution or in the first quintile (Q1, the poorest ones) compared to the individual at the highest point of the income distribution or the last income quintile (Q5, the richest ones).

Logistic regression was used to calculate RII with same relative income rank previously calculated, which was input into the regression as an independent ordinal variable. The regression coefficient and the standard error were used to calculate the odds ratio (OR) with 95 % confidence interval. This OR is known as RII. The results were interpreted as the comparison of the extremes, with the difference between Q1 and Q5 and SII referring to the absolute inequality as percentage points, while the ratio between Q1 and Q5 and RII expressed the relative inequality based on the chance of the individual having that outcome. The greater SII and/or RII, the greater the level of inequality in the socioeconomic hierarchy [16].

The inequality observed between income and the incidence of LBW and SGA in the four cohorts was classified as bottom pattern or bottom inequality (when the prevalence among the poorest is positioned very far from the prevalence among other economic groups), top pattern or top inequality (when prevalence among the richest is positioned very far from the prevalence among others) or linear pattern (similar distance among the groups analyzed) [2].

### Data analysis

The analyses were restricted to single live births since several outcomes, such as intrauterine growth restriction, did not include stillbirths or would be repeated data from the same family in the case of twins.

The chi-squared test (*X*^2^) was used to compare the distribution of maternal characteristics in the four cohorts and, when possible, *X*^2^ for linear trend was calculated.

The independent contribution of household income was determined in each of the outcomes analyzed, through the inclusion in the final model, the adjusted analysis, of the variables skin color, schooling, mother’s age, and mother’s marital status. All analyses were performed using the software Stata 13.1.

## Results

Table 1 summarizes the main frequencies of the outcome and exposure variables assessed in each cohort. The prevalence of LBW increased (8.2 to 10.9 %) while SGA decreased (14.4 to 9.2 %) over the period studied. Mother’s white skin color prevailed in the four studies and the number of black or mixed-color mothers increased over the years. Mother’s schooling increased and the percentage of adolescent mothers in 2011 are comparable to that in 1993 after an increase in 2004. The number of women living with a partner increased from 8.2 to 16.4 % (2004 cohort) and later decreased to 13.3 % in 2011 (Table 1).

**Table 1:** Maternal and child characteristics in four birth cohort studies in Pelotas

Variables	Pelotas 1982 n (%)	Pelotas 1993 n (%)	Pelotas 2004 n (%)	Pelotas 2011 n (%)	p (*X* ^2^)	p (*X* ^2^) trend
Birthweight						
≥2500 g	5333 (91.8)	4683 (90.9)	3772 (91.0)	5457 (89.1)	<0.001	<0.001
<2500 g	478 (8.2)	468 (9.1)	372 (9.0)	666 (10.9)		
Small for the Gestational Age						
No	3931 (85.6)	3861 (84.1)	3320 (88.6)	5560 (90.8)	<0.001	<0.001
Yes	659 (14.4)	729 (15.9)	450 (11.9)	563 (9.2)		
Ethnic origin						
White	4773 (82.1)	3996 (77.4)	3030 (73.1)	4132 (67.5)^a^	<0.001	<0.001
Black/mixed	1040 (17.9)	1170 (22.6)	1117 (26.9)	1986 (32.5)^a^		
Family income (quintiles)						
1st (poorest)	1159 (19.8)	1037 (20.1)	846 (20.4)	1198 (19.6)	0.016	0.18
2nd	1166 (20.1)	1161 (22.5)	841 (20.2)	1349 (22.0)		
3rd	1166 (20.1)	922(17.8)	802 (19.4)	1224 (20.0)		
4th	1162 (20.0)	1029 (19.9)	846 (20.4)	1215 (19.8)		
5th (better off)	1163 (20.0)	1019 (19.7)	812 (19.6)	1138 (18.6)		
Maternal schooling (y)						
0–4	1922 (33.1)	1441(27.9)	639 (15.6)	514 (8.4)	<0.001	<0.001
5–8	2425 (41.7)	2392 (46.3)	1691(41.1)	2316 (37.8)		
9–11	646 (11.1)	911 (17.7)	1362 (33.2)	2149 (35.1)		
≥12	816 (14.1)	417 (8.1)	414 (10.1)	1144 (18.7)		
Age (y)						
<20	908 (15.6)	910 (17.6)	792 (19.1)	1065 (17.4)	<0.001	0.09
20–34	4339 (74.6)	3692 (71.5)	2800(67.6)	4243 (69.3)		
≥35	568 (9.8)	565 (10.9)	553(13.3)	816 (13.3)		
Marital status						
With partner	5336 (91.8)	4528 (87.7)	3468 (83.6)	5297 (86.5)	<0.001	<0.001
Single mother	475 (8.2)	640 (12.4)	679 (16.4)	827 (13.5)		

^a^Self-reported variable; y = years; *X* ^2^ = qui-square

The highest prevalences of LBW and SGA were observed in those families with the lowest income in the four studies analyzed (Table 2).

**Table 2:** Prevalence of delivery outcomes (low birthweight and small for the gestational age) according to family income quintiles among four birth cohort studies in Pelotas

Outcomes	Pelotas 1982					Pelotas 1993					Pelotas 2004					Pelotas 2011				
	1st^a^	2nd	3rd	4th	5th	1st^a^	2nd	3rd	4th	5th	1st^a^	2nd	3rd	4th	5th	1st^a^	2nd	3rd	4th	5th
	*p* <0.001					*p* = 0.006					*p* <0.001					*p* =0.003				
Low Birthweight (%)	13.5	8.6	6.9	6.6	5.7	10.6	10.2	9.2	9.1	6.2	13.1	10.5	6.6	7.5	7.0	13.0	12.1	10.5	10.3	8.3
	*p* <0.001					*p* <0.001					*p* <0.001					*p* = 0.003				
Small for the Gestational Age (%)	22.3	15.8	14.3	13,1	8.2	19.5	16.2	17.5	14.6	11.9	15.7	14.7	12.0	8.9	8.8	11.5	9.6	9.6	7.8	7.3

^a^Poorest

Inequalities were observed in LBW and SGA through the analysis of SII and adjusted SII (ASII) regarding skin color, schooling, and mother’s marital status and age. The adjusted analysis showed that the greatest absolute income inequality in LBW due to income was observed in 1982 from the difference between the poorest (Q1) and the richest (Q5), which was 8.5 percentage points (PP). This index decreased to 1.3 PP in 1993, increased again in 2004, and dropped in 2011, reaching 1.8 PP (Table 3). In SGA, ASII behaved similarly to that in LBW, and the 1982 study had the greatest absolute inequality (14.4 PP) and greater differences in subsequent years compared to LBW (Table 3).

**Table 3:** Crude and multivariable associations of family income (quintiles) with delivery outcomes among four birth cohort studies in Pelotas

	Models	Pelotas 1982	Pelotas 1993	Pelotas 2004	Pelotas 2011
		Slope index of inequality: absolute difference in health status between those at the bottom and those at the top of the income hierarchy (95 % CI)			
Low Birthweight	Raw	0.088 [0.063, 0.113]	0.049 [0.022, 0.077]	0.077 [0.0463, 0.108]	0.056 [0.028, 0.083]
	Adjusted (*)	0.085 [0.049, 0.121]	0.013 [-0.018, 0.045]	0.059 [0.022, 0.095]	0.018 [-0.015, 0.050]
Small for the Gestational Age	Raw	0.153 [0.117, 0.189]	0.084 [0.046, 0.121]	0.099 [0.062, 0.136]	0.050 [0.025, 0.076]
	Adjusted (*)	0.144 [0.092, 0.197]	0.060 [0.017, 0.103]	0.071 [0.028, 0.114]	0.048 [0.018, 0.078]
		Relative index of inequality: OR for each outcome comparing those at the bottom to those at the top of the income hierarchy (95 % CI)			
Low Birthweight	Raw	3.29 [2.34, 4.63]	1.83 [1.30, 2.56]	2.60 [1.77, 3.82]	1,78 [1.33, 2.37]
	Adjusted (*)	3.06 [1.89, 4.95]	1.18 [0.81, 1.72]	2.09 [1.33, 3.27]	1.20 [0.86, 1.68]
Small for the Gestational Age	Raw	3.49 [2.59, 4.72]	1.88 [1.41, 2.49]	2.59 [1.82, 3.70]	1.83 [1.35, 2.50]
	Adjusted (*)	3.12 [2.04, 4.79]	1.57 [1.14, 2.16]	1.98 [1.31, 2.98]	1.78 [1.24, 2.55]
Small for Gestational Age	Crude	3.49 (2.59, 4.72)	1.88 (1.41, 2.49)	2.59 (1.82, 3.70)	1.83 (1.35, 2.50)
	Adjusted	3.12 (2.04, 4.79)	1.57 (1.14, 2.16)	1.98 (1.31, 2.98)	1.78 (1.24, 2.55)

(*) adjusted for ethnic origin, schooling, marital status, and maternal age

The RII observed in all birth cohorts showed that the poorest mothers had higher chances of having NN with LBW or SGA compared to the richest mothers (Table 3). The effect adjusted for confounding factors, in most cases, decreased the magnitude of all outcomes studied not changing the direction of the association, remaining the poorest ones at higher risk of LBW or SGA outcomes (Table 3).

A downward trend was found in the income inequalities between the cohorts from the 1980s and 1990s, with an increase in 2004 and a new reduction in 2011 for both outcomes studied (Table 3).

**Fig. 1:**
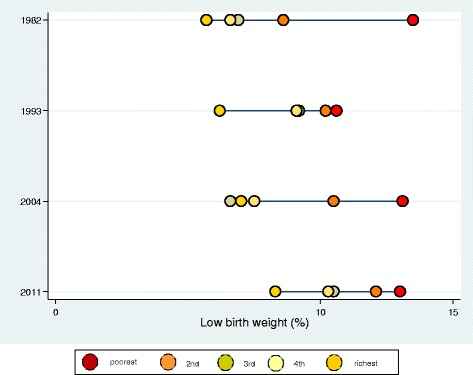
Inequality for delivery outcome low birthweight among neonates from four birth cohort studies in Pelotas

**Fig. 2:**
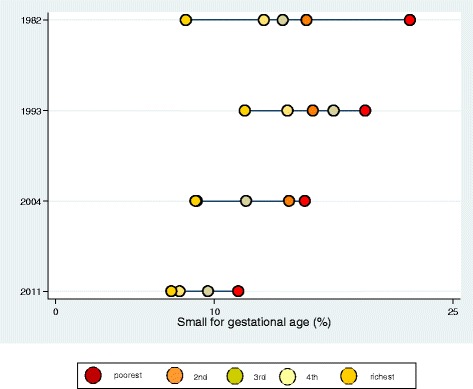
Inequality for delivery outcome small for the gestational age among neonates from four birth cohort studies in Pelotas

In LBW, the bottom-pattern inequality was pronounced in 1982 and 2004, while the top-pattern inequality was observed in 1993 and 2011 (Fig. 1). Among the SGA, important inequalities was found in the top and bottom patterns in 1982; the top pattern was maintained in 1993 (almost linear) and 2004; and a linear pattern was found in 2011 (Fig. 2). No downward trend was found in the prevalence of LBW among the poorest individuals, however, such trend was observed in the SGA in all four cohorts, associated with the reduction in inequalities over the last decades (Figs. 1 and 2).

## Discussion

### Statement of principal findings

Important income inequalities were found in the four studies regarding LBW and SGA. Although birthweight reflects fetal growth and despite the use of birthweight below 2,500 g as an important proxy for intrauterine growth in public health, the index does not reflect the fetus’s holistic growth. One of the great shortcomings in using it by itself is the fact it does not differentiate whether the weight is low because of newborn birth earlier than expected (for example, preterm birth) or because there were restrictions in the fetus’ development and the infant small size for the gestational age [5, 17].

The mother’s unfavorable socioeconomic level, based on income, schooling, or other sociodemographic factors, can favor NN with LBW or SGA [4–6]. Previous studies already showed a similar association of low income with LBW in Canada [18] and with LBW and SGA in the United States [19], Brazil [20, 21], and Indonesia [22].

Low income can be an important socioeconomic factor related to the social exclusion of the individual in the community and, therefore, reflects great inequalities in health [6]. Mother’s or family’s income impacts mother and child health in several ways, such as pregnant woman’s, good nutritional quality, access to healthcare for proper prenatal care, and early identification of morbidities specific to gestation that may lead to preterm birth and/or IUGR [22–24].

Even after adjusting for sociodemographic factors that predispose to IUGR, the increased risk of LBW and SGA remains in low-income families. It must be pointed out that mothers born with LBW or SGA have higher chances of having children with the same characteristics, which helps perpetuate inadequate neonatal outcomes along with the poverty cycle^.^ [25, 26].

It has already been shown that gains in income, schooling, and occupation may impact health inequalities at different aggregation levels with consequences in better individual and community health [2, 3, 26, 27]. Over the last 30 years, intense changes have occurred in public policies in Brazil that were related to socioeconomic factors:

i. Progress in reducing Global Hunger Index in Brazil, which dropped by 52.5 % in 2009 compared to 1990, with an important trend of reducing malnourishment in the population (particularly among children), and of decreasing micronutrient deficit and, consequently, anemia, hypovitaminosis, and malnourishment of future mothers, despite the slight impact on preterm births and IUGR [24, 28];
ii. Considerable drop in poverty and extreme poverty rates, from 41 and 17.8 % in 1982 to 15.9 and 5.3 % in 2012, respectively [29];
iii. Drop of the GINI coefficient from 0.591 in 1982 to 0.530 in 2012 [29];
iv. Increase in the Human Development Index (HDI), while Brazil is considered a country with high human development: HDI of 0.744 (2013), life expectancy at birth of 73.9 years (2013), mean schooling of 7.2 years (2013), and gross national income (GNI) *per capita* of USD 14,275 (2011) [30].

### Meaning of the study: possible mechanisms and implications for clinicians or policymakers

All these socioeconomic changes might have impacted the results presented. Relevant aspects in mother and child policies must still be prioritized in order to reduce neonatal mortality, particularly early mortality, in which LBW and/or gestational age below 37 weeks are still important risk factors [23, 26, 28]. In 2011, the Brazilian Ministry of Health committed to changing the model of delivery and birth care, created a healthcare network called “Rede Cegonha” (Stork Network), which features multidisciplinary teams comprising both physicians and non-physicians and uses protocols and indicator monitoring. This model has already been successfully used in several countries to reduce neonatal morbidity-mortality and represents a highly valuable outlook in perinatal care [31].

One of the relevant aspects in the present study concerns the reduction in socioeconomic inequality by analyzing income along with the drop in the prevalence of SGA over the last 30 years. That strengthens the proposals of the programs recommended by major international institutions such as the World Health Organization (WHO) and the United Nations Children’s Fund (UNICEF), among others, regarding actions that foster the improvement in parental schooling and/or professional training so that they have better chances in the work market and, consequently, better income and socioeconomic situation [2, 25, 26].

The reduction of children with IUGR alone, whether preterm or not, would already lead to short-term benefits such as fewer neonatal sequelae and longer life expectancy and, in the long term, lower comorbidity indices in the population. Longitudinal studies on LBW support this proposal since they suggest a potential risk for future cardiometabolic diseases such as high blood pressure, type-II diabetes mellitus, and coronary disease, particularly in former SGA [25, 26].

### Strengths and weaknesses of the study

The large amount of information collected in this study from birth cohorts from the same Brazilian city enabled knowing and comparing socioeconomic and demographic aspects of that population to evaluate the association of inequalities with LBW and SGA.

One of possible limitations in this study is the fact that different methods were used to determine gestational age over the time periods: Dubowitz’s method (1982 and 1993), by a combination of LMP, ultrasound (when available) and by Dubowitz’s method (2004) and by a combined of LMP, ultrasound and neonate measures using an internationally standard chart (2011). The use of combined methods could increase the prevalence of SGA due to greater accuracy. However, this was not observed as in the last two studies. Where gestational age was better evaluated, SGA prevalence was lower than in the first ones.

### Unanswered questions and future research

The goal of the study was not to follow these individuals with poor neonatal outcomes for greater evolutionary insight on neonatal and child morbidity-mortality. That could contribute to the assessment of to what extent the inequalities may impact and increase the subsequent risks in childhood for these important public health indicators. These could be future questions to be answered in a later analysis of the subsequent data.

## Conclusion

In summary, important income inequalities were identified in the incidence of LBW and SGA in the four studies, with a greater incidence of all outcomes among the poorer individuals. Over time, despite the oscillations, a reduction in absolute and relative inequality was observed in LBW and SGA.

These results highlight the importance of public health policies for support and social inclusion, for income improvement, or for other factors that positively impact in the socioeconomic position of the families at major vulnerability. Moreover, the full implementation of specific programs that contemplate the early detection of IUGR and the consequent intervention (when possible) among the economically underprivileged populations may be key factors to reduce the prevalence of LBW and SGA, which are still relevant in low- and medium-income countries.

## Abbreviations

ASII, adjusted slope index of inequality; CRL, crown–rump length; IUGR, Intrauterine growth; LBW, low birthweight; LMP, last menstrual period; NN, restriction neonates; OR, odds ratio; RII, relative index of inequality; SGA, small for the gestational age; SII, slope index of inequality
